# Subacute Myocardial Rupture Following Tirofiban Treatment

**DOI:** 10.5812/cardiovascmed.10065

**Published:** 2013-07-31

**Authors:** Özgür Çiftçi, Murat Günday, Tonguç Saba, Mehmet Özülkü

**Affiliations:** 1 Department of Cardiology, Faculty of Medicine, Baskent University, Ankara, Turkey; 2 Department of Cardiovascular Surgery, Faculty of Medicine, Baskent University, Ankara, Turkey

**Keywords:** Tirofiban, Rupture, Emergencies

## Abstract

A 74-year-old male patient was admitted to our emergency department with post-MI angina. On account of the anginal complaint that continued for three days, a coronary artery angiography was undertaken. A percutaneous transluminal coronary angioplasty was performed, followed by the implantation of a coronary stent, and coronary perfusion (TIMI-3) was achieved in the left anterior descending artery. Medical treatment (with acetylsalicylic acid, clopidogrel, metoprolol, atorvastatin and enoxaparine) and tirofiban infusion were duly administered in the coronary care unit. After twenty-four hours, however, acute dyspne, hypotension and tachycardia developed, making it necessary to perform an echocardiography. Since the echocardiography revealed a frank pericardial effusion, the patient was immediately taken to the operation room. The ventricular free wall rupture was repaired with Surgicel, which was prepared in three layers and fixed to the myocardium by tissue glue; cardiopulmonary bypass was not used. To our knowledge, our study constitutes the first case report of a tirofiban-induced free wall rupture.

## 1. Introduction

The left ventricular free wall rupture, although rarely encountered, constitutes one of the most fatal complications of acute myocardial infarction. Surgical repair is mandatory, even though operative mortality is high. Early diagnosis and surgical repair are crucial in treatment. However, it remains controversial which is the most suitable method of surgical management. Glycoprotein IIb/IIIa antagonists like abciximab, eptifibatide and tirofiban, the last a nonpeptide tyrosine derivative (Aggrastat, Merck & Co), have generally demonstrated good clinical benefits and safety profiles in acute coronary syndrome. But we present in this study a complication of subacute myocardial infarction, involving ventricular free wall rupture that developed after the administration of tirofiban. Surgical treatment was administered through the implantation of Surgicel (Ethicon, Inc., a Johnson & Johnson company; Somerville, NJ), which was fixed in place with tissue glue.

## 2. Case Report

**Figure 1.**
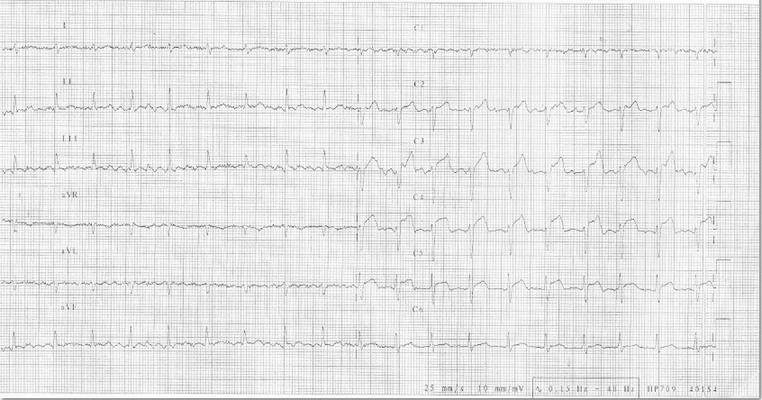
A ST-segment Elevation is Observed in the Anterior Derivation

**Figure 2.**
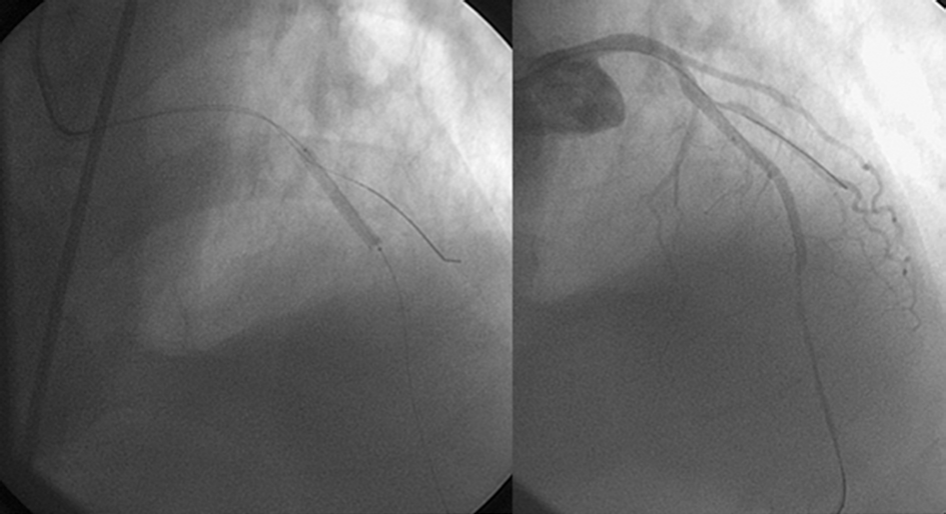
a- The left Anterior Descending Artery as Seen During the Angioplasty. b- The Left Anterior Descending Artery as Seen After the Angioplasty

**Figure 3.**
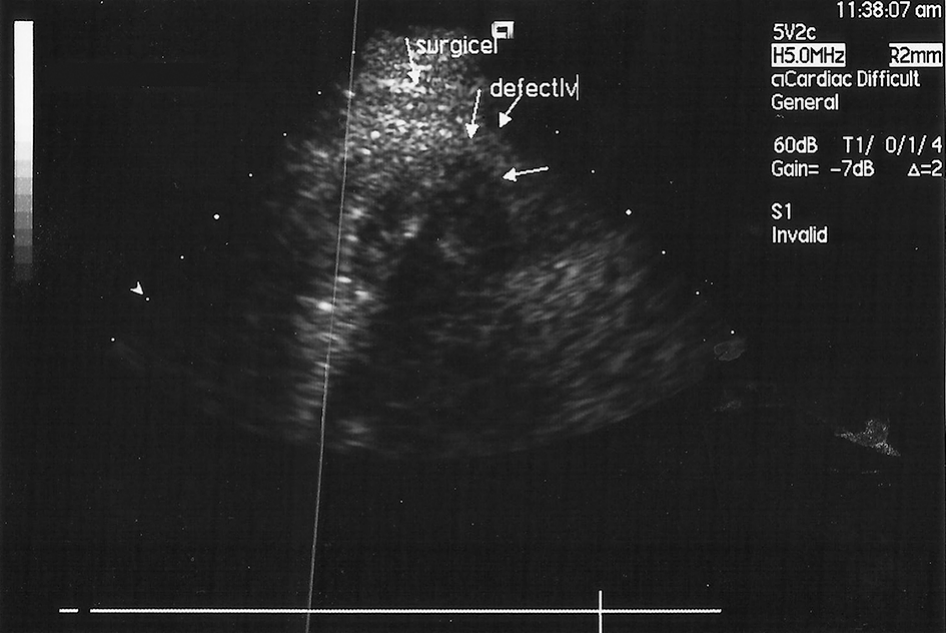
The Control Echocardiography Did not Reveal any Pericardial Effusion, and the Implanted Surgicel Was Documented as Well

A 74-year-old male patient was admitted to our emergency department with post-MI angina. An electrocardiogram showed ST elevations in the anterior derivation (Figure 1). The first cardiac enzymes were high (CKMB: 224 (< 24), TnI:17.1(< 1)). The patient had diabetes mellitus, chronic obstructive lung disease and smoking in his medical history. He was admitted to our coronary care unit. An echocardiography revealed anterior and apical wall akinesia, while there was no sign of pericardial effusion. Because of the persisting anginal complaint, which continued for three days, a coronary artery angiography was performed. The angiography demonstrated that there was 99% occlusion in the mid-left anterior descending artery (LAD), as well as a muscular bridge that caused 80% occlusion in the distal LAD. On the other hand, the circumflex and right coronary arteries were normal. Eight units of clopidogrel were administered before applying angioplasty. Subsequently a percutaneous transluminal coronary angioplasty was performed, followed by the implantation of a 2.75x22 mm coronary stent, and coronary perfusion (TIMI-3) was achieved in the distal LAD (Figure 2). We did not encounter any complications during the procedure. Medical treatment with acetylsalicylic acid, clopidogrel, metaprolol, atorvastatin, and enoxaparine, as well as tirofiban infusion –with the dose regimen adjusted according to the chart for PCI– were duly administered in the coronary care unit. During the patient’s follow-up, however, there was an onset of acute dyspne, hypotension and tachycardia, developing 24 hours after the angioplasty. The ECG revealed that the QRS gain had decreased and there was tachycardia in all derivations. In the echocardiography that was subsequently performed, a frank pericardial effusion was detected, with apical wall motion abnormalities, and cardiac tamponade physiology was observed in Doppler flow velocities (with right atrial systolic collapse, right ventricular diastolic collapse, a dilated inferior vena cava and so on). Repeated coronary angiography revealed that the flow was normal in the LAD. The patient was immediately taken to the operation room, and the heart was approached through sternotomy. After opening the pericardium, the accumulated blood and clots were removed. Then the ventricular free wall rupture was repaired with Surgicel, which was prepared in three layers and fixed to the myocardium by tissue glue; cardiopulmonary bypass was not used. Following the operation, hemodynamic stability was achieved and control echocardiography did not reveal any pericardial effusion; the implanted Surgicel was documented as well (Figure 3). 

## 3. Discussion

Ventricular free wall rupture is a leading cause of death after acute myocardial infarction (AMI), and early diagnosis and treatment are very important. It is encountered in about 3% of the cases in AMI (1), and usually occurs within the first two weeks following the infarction. From the point of view of pathophysiology, it may be classified as acute, subacute or chronic. Although acute myocardial rupture is almost universally fatal, subacute rupture may prove amenable to expedient surgery with hemorrhage control and myocardial repair. Echocardiography has been used as a diagnostic tool for suspected myocardial rupture and prompt diagnosis in patients with this second kind of rupture (2). The main echocardiographic findings in the patients in question are pericardial effusion with thickness > 10 mm, intrapericardial echoes, and signs of tamponade (right atrial and ventricular collapse or the actual tear itself may be observed). Yet another useful diagnostic method is obtaining an uncoagulated blood sample through pericardiocentesis. Several different surgical techniques have been proposed for treatment. The traditional technique consists of infarctectomy and approximating the free edges with interrupted mattress sutures with felt or pledges. Recently, the use of epicardial patch repair without extracorporeal circulation has been increasing in popularity (3). GP IIb/IIIa inhibitors include abciximab, eptifibatide and tirofiban. Tirofiban is a highly selective inhibitor of GPIIb/IIIa platelet receptors in humans. Glycoprotein IIb/IIIa inhibitors reduce the morbidity and mortality linked to platelet activation. However, adverse events involving thrombosis or bleeding have also been reported in cases of therapy with glycoprotein IIb/IIIa antagonists (4). Although it seems likely that the perforation in our case was caused or precipitated by tirofiban, it is also possible that a free rupture, unrelated with tirofiban and linked to the recent MI, occurred coincidentally after our treatment. Other possibilities, such as an adverse effect of the anticoagulant regiment, should also be taken into consideration.

## References

[1] Mochizuki T, Kawaue Y, Imura I, Wada S, Tsuchiya T (1989). [A study of left ventricular rupture associated with acute myocardial infarction].. Nihon Kyobu Geka Gakkai Zasshi..

[2] Coletti G, Torracca L, Zogno M, La Canna G, Lorusso R, Pardini A (1995). Surgical management of left ventricular free wall rupture after acute myocardial infarction.. Cardiovasc Surg..

[3] McMullan MH, Maples MD, Kilgore TL, Jr, Hindman SH (2001). Surgical experience with left ventricular free wall rupture.. Ann Thorac Surg..

[4] Panduranga P, Sulaiman K (2011). Severe thrombocytopenia following tirofiban infusion.. Indian J Pharmacol..

